# Use of social network as a coping strategy for depression among young people during the COVID-19 lockdown: findings from the COMET collaborative study

**DOI:** 10.1186/s12991-022-00419-w

**Published:** 2022-11-14

**Authors:** Laura Orsolini, Umberto Volpe, Umberto Albert, Claudia Carmassi, Giuseppe Carrà, Francesca Cirulli, Bernardo Dell’Osso, Valeria Del Vecchio, Marco Di Nicola, Vincenzo Giallonardo, Mario Luciano, Giulia Menculini, Maria Giulia Nanni, Maurizio Pompili, Gabriele Sani, Gaia Sampogna, Alfonso Tortorella, Andrea Fiorillo

**Affiliations:** 1 Clinical Psychiatry Unit, Department of Clinical Neurosciences/DIMSC, School of Medicine, Università Politecnica delle Marche, Via Tronto 10/A, 60126 Ancona, Italy; 2 Department of Medicine, Surgery and Health Sciences, University of Trieste, Department of Mental Health, Azienda Sanitaria Universitaria Giuliano Isontina—ASUGI, Trieste, Italy; 3 Department of Clinical and Experimental Medicine, University of Pisa, Pisa, Italy; 4 Department of Medicine and Surgery, University of Milan Bicocca, Milan, Italy; 5 National Institute of Health, Rome, Italy; 6 Department of Biomedical and Clinical Sciences Luigi Sacco, Aldo Ravelli Center for Neurotechnology and Brain Therapeutic, University of Milan, Milan, Italy; 7 Department of Psychiatry, University of Campania “L. Vanvitelli”, Naples, Italy; 8 Department of Neuroscience, Section of Psychiatry, University Cattolica del Sacro Cuore, Rome, Italy; 9 Department of Psychiatry, University of Perugia, Perugia, Italy; 10 Department of Neurosciences and Rehabilitation, University of Ferrara, Ferrara, Italy; 11 Department of Neurosciences, Mental Health and Sensory Organs, Faculty of Medicine and Psychology, Sapienza University of Rome, Rome, Italy; 12 Fondazione Policlinico A. Gemelli IRCCS, Rome, Italy

**Keywords:** Aggressiveness, COVID-19, Impulsiveness, Problematic social media use, Social networking

## Abstract

**Background:**

Use of social media (SM) has exponentially grown particularly among youths in the past two years, due to COVID-19-related changing lifestyles. Based on the Italian COvid Mental hEalth Trial (COMET), we investigated the association between SM use and depressive symptoms among Italian young adults (aged 18–24).

**Methods:**

The COMET is a nationwide multi-center cross-sectional study that investigated socio-demographic data, social networking addiction (BSNAS), depression, anxiety, and stress (DASS-21), as well as impulsiveness (BIS-15) and aggressiveness (AQ) in a large sample of youngsters, in order to assess the association between BSNAS and DASS-21 indices. Mediation analyses were performed to evaluate the role of impulsiveness and aggressive personality traits in the association between SM use (SMU) and depression.

**Results:**

75.8% of the sample (*n* = 491) had a problematic SMU. SMU was reduced by high AQ and high DASS-21 scores (*F* = 42.338, *p* < 0.001, *R*^2^ = 0.207). Mediation analyses showed that SMU negatively predicted depressive symptomatology with the interaction mediated by AQ total (*ß* = − 0.1075), physical (*ß* = − 0.207) and anger (*ß* = − 0.0582), BIS-15 total (*ß* = − 0.0272) and attentional (*ß* = − 0.0302). High depressive levels were predicted by high AQ scores, low SMU levels, low verbal and physical AQ, and low attentional BIS-15 (*F* = 30.322, *p* < 0.001, *R*^2^ = 0.273). Depressive symptomatology negatively predicted SMU with their interaction mediated by AQ total (*ß* = − 0.1640), verbal (*ß* = 0.0436) and anger (*ß* = − 0.0807), BIS-15 total (*ß* = − 0.0448) and attentional (*ß* = − 0.0409).

**Conclusions:**

SMU during the early phases of the COVID-19 pandemic could have a beneficial role in buffering negative consequences linked to social isolation due to quarantine measures, despite this association being mediated by specific personality traits.

## Introduction

Social Media (SM) is a group of Internet-based applications that build on the ideological and technological foundations of web 2.0. (i.e., the way in which end-users started to utilize the Internet platform, whereby content and applications are no longer created and published by individuals but instead continuously modified by all users in a participatory and collaborative way) and that allow the creation and exchange of user-generated content [1–3]. SM platforms have exponentially grown in their use over the last decade, particularly among young people [4, 5] and the so-called ‘digital natives’ have grown up immersed in the digital technology, with an habitual SM use of about 90% [6, 7].

Several studies outlined the positive role of SM in fostering individual’s wellbeing, by enriching everyday-user experiences, facilitating social cohesion, increasing feelings of connectedness and closeness with friends, as well as enhancing selective self-disclosure and social support [8–11]. An enhanced friendship quality and perceived increased social support, due to the social networking use, were also identified as protective factors against depressive symptomatology [12, 13]. SM use is becoming increasingly common also in mental health care, by facilitating networking with peers/colleagues/patients, mentorship, education and research, particularly among youths [14]. However, despite these promising beneficial advantages, other studies raised concerns regarding the potential addictive use of social networking platforms, by underlining the negative association between a problematic social media use (PSMU) and psychological consequences [15–19]. PSMU is characterized by an excessive individual’s concern about online social networking activities, being driven by a strong motivation to log on or use SM, and devoting so much time and effort to use these online platforms. This may lead the subject to totally or partially impair other social activities, including the school/university/job, interpersonal relationships and overall wellbeing [15, 16]. The global pooled prevalence of PSMU was estimated to be 17.4% (95%CI 12.4–23.9), considering the increase occurred due to the COVID-19 [20]. PSMU, including the compulsive involvement in online social networking activities, has been also negatively associated with worst individual's physical and mental health, poor wellbeing, relationships, reduced self-esteem levels, poor sleep quality, increased loneliness and depressive levels [17, 21–26], particularly among adolescents and young adults [27–29]. Systematic reviews reporting a suggestive detrimental effect of wireless devices and SM use on youth’ mental health also recommended caution in interpreting these findings, because of the lack of high-quality longitudinal studies [30–32]. Recent literature also reported that the effect on youth mental health mainly depends on the time and type of SM used as well as on individual vulnerability [31–33].

The rapid outbreak of the coronavirus pandemic (COVID-19) had a significant impact on people’s everyday life, as restrictive measures were imposed to reduce physical contact to limit the viral transmission [34–37]. These changes in lifestyle were associated to an increase of the frequency and intensity of individual’s engagement in online activities, including social networking use [20, 38]. Such an increase has been associated to managing the reduced sense of control, to escaping the anxiety and uncertainty feelings and to being constantly informed about updates and news and maintain peer-to-peer social contact [39, 40].

According to our previous results from the COMET study [41, 42], COVID-19-related social isolation predispose to increased problematic internet activities (i.e., videogaming and internet addiction) in the general population, while youngsters reported a higher increase in SM activities compared to adults [43]. The COVID-19-related ‘stay-at-home’ policy may have disrupted certain social needs (i.e., need for relatedness) and incentivized the SM use, to gain social compensation through virtual interactions [44, 45]. Other studies documented that SM use and relatedness need satisfaction improve mental health issues, particularly depressive symptoms and loneliness [46–49].

In order to investigate the role of social networking use during the COVID-19 outbreak among more vulnerable individuals (i.e., young people), we carried out a post hoc analysis study by selecting only the younger population (aged 18–24; [50]), from the COMET study sample [43]. We aimed to focus on the association between the psychopathological burden and the SM use during the COVID-19 lockdown. The main objectives of the present study were to: (a) report the characteristics of young people experiencing PSMU versus non-PSMU, during the phase II and phase III of COVID-19 pandemic in a multi-center sample of young adults aged 18–24 years; (b) describe the effects of the COVID-19-related psychopathological symptoms on the emergence of PSMU in the same sample; (c) evaluate the specific role of SM use during the COVID-19 outbreak in the relationship between PSMU and depressive symptomatology.

## Materials and methods

The COvid Mental hEalth Trial (COMET) is a population-based Italian multi-center study, with cross-sectional observational design, using a snowball sampling method. The full study protocol is available elsewhere [41, 42]. All participants provided their written informed consent to participate in the study. The study was approved by the Ethical Review Board of the University of Campania “L. Vanvitelli” (Protocol number: 0007593/i) and it complies with the Declaration of Helsinki.

Eligible participants were requested to complete all sections of the survey, including a socio-demographic section, clinical data (e.g., pre-existing physical and/or mental illness, information regarding the COVID-19 infection and hospitalization, isolation and/or quarantine, and so forth) and a set of validated questionnaires (see “Measures”). For the full protocol and methodology of the COMET satellite study on web-based psychopathologies, see Volpe et al. [43].

### Measures

The *Depression, Anxiety and Stress Scale (DASS-21, Italian short version*) [51] is a self-report questionnaire consisting of 21 items, on a 4-point Likert scale, divided in three subscales (depressive, anxiety and stress). The DASS-21 total score provides a general psychopathology index [52]. Here, we focused on the “depression” subscale dimension measures the level of dysphoria, hopelessness, devaluation of life, self-deprecation, and lack of interest/involvement, anhedonia and inertia.

The *Bergen Social Networking Addiction Scale* (*BSNAS, Italian version*) [53] is a 6-item self-report questionnaire on a 5-point Likert scale assessing the SM use over a period of 12 months. BSNAS items measure each core addiction element (i.e., salience, mood modification, tolerance, withdrawal, conflict and relapse). Higher BSNAS scores indicate a greater risk of SM addiction. The BSNAS demonstrated a good reliability [54]. Based on the BSNAS cut-off of 16, the sample was split in two groups, to discriminate between those participants with a problematic social networking use (BSNAS+) versus those participants without a problematic social networking use (BSNAS−) [53].

The *Barratt Impulsiveness Scale (BIS-15*, *Italian version*) [55] is a short-version of the self-reported Barratt Impulsiveness Scale (BIS) [56], consisting of 15 items on a 4-point Likert scale and three subscales: motor (acting without thinking), attentional (a lack of focus on the ongoing task) and nonplanning impulsivity (orientation to the present rather than to the future) ones [57]. The severity and type of impulsiveness were tested for their potential mediator roles in the relationship between BSNAS and DASS-21 depression.

The *Aggression Questionnaire (AQ, Italian version*) [58] is a 29-item self-report questionnaire on a 7-point Likert scale, developed to assess four major components of aggression (physical aggression, verbal aggression, anger and hostility). The severity and type of impulsiveness were tested for their potential mediator roles in the relationship between BSNAS and DASS-21 depression.

### Statistical analysis

Descriptive statistics were performed in order to investigate the socio-demographic and clinical characteristics of the sample. Categorical variables are summarized as frequency (*n*) and percentage (%). The *χ*^2^ test was used to compare all categorical variables with each other as well as to compare all socio-demographic variables between two groups (i.e., BSNAS + vs BSNAS −). Continuous variables are summarized as mean and standard deviation (SD). The normality of the distribution of the continuous variables were tested using the Kolmogorov–Smirnov test. Since our continuous variables showed a normal distribution, independent samples Student’s *T*-test and two-way tailored analysis of variance (ANOVA) were used to investigate the level of severity of PSMU (as measured by BSNAS), according to a set of categorical independent variables (i.e., gender, phase II vs. phase III, being infected and/or hospitalized due to COVID-19 infection, being isolated due to COVID-19 infection, being isolated due to a contact with someone affected with COVID-19, having lost job due to the COVID-19 pandemic, having a pre-existing physical disorder, having a pre-existing mental disorder).

Bivariate Pearson’s correlations have been used to investigate potential relationships between BSNAS total score and the following continuous variables: DASS total score, DASS depressive symptoms, DASS anxiety symptoms, DASS stress symptoms, AQ total score and subscales, and BIS-15 total score and subscales. Statistical analyses were firstly carried out by considering BSNAS (as measure of PSMU) as primary outcome in order to evaluate whether COVID-19-related variables could be predictors of a higher risk of developing PSMU in our cohort of young people recruited during the second and third Italian COVID-19 waves. Then, we carried out all analyses by considering as primary outcome the DASS-21 depressive symptomatology, in order to identify whether PSMU and/or higher vs. lower levels of BSNAS could be risky or protective factors for the emergence of clinically significant depressive levels in our cohort of young people during the COVID-19 pandemic.

Therefore, in order to identify possible predictors of the levels of PSMU (as measured by BSNAS), a multivariate linear regression model was performed, including as independent variables: DASS-21 total score, DASS-21 depression, anxiety, and stress subscales, gender, having being infected by the COVID-19, having being isolated due to the contact with a person infected by the COVID-19, having being quarantined due to the infection with COVID-19, having a pre-existing mental disorder and/or having a pre-existing physical disorder. In addition, a stepwise binary logistic regression analysis was performed in order to evaluate the predictors associated with the presence of PSMU (vs. non-PSMU) after categorizing BSNAS total score into two dichotomous values according to the established cut-off of 16. The estimated odds ratios (OR) along with the 95% of confidence intervals (95% CI), and standardized coefficient *β* values were generated for each variable.

Furthermore, in order to evaluate possible predictors of the depressive levels (as measured by the depressive subscale of the DASS-21) in our sample of young people, a multivariate linear regression model was performed, including as independent variables all COVID-19-related variables (as above described), AQ total and subscales, BIS-11 and subscales, BSNAS total score and dichotomous BSNAS variable (i.e., presence vs. absence of PSMU). Following the identification of significant predictors of depressive symptomatology, PROCESS macro (version 3.5.3, February 2021) for SPSS [59] was run to carry out mediation analyses (Model 4) to test whether the direct or indirect effect of BSNAS (as independent variable) on DASS-21 depression subscale (as dependent variable), were mediated by AQ total score and/or subscales and BIS-11 total score and/or subscales. Indicators of indirect effects were tested using a bias-corrected bootstrapping (*n* = 5000) with 95% CI, by setting a statistical significance when the 95% CI does not contain zero. For all analyses, the level of statistical significance was set at *p* < 0.05, two-tailed. All analyses were performed using the software Statistical Package for Social Science (SPSS) version 27.0 (IBM SPSS Statistics, Chicago, IL, United States).

## Results

### Socio-demographic and psychopathological characteristics

The final sample consists of 491 individuals, with a slight prevalence of females (64.4%, *N* = 316) and single (58%, *N* = 285). The mean age was 21.8 (SD = 1.7) years, without any statistical significant gender-based differences. The most frequent educational levels were university degree (53%; *N* = 260) and high school diploma (46%; *N* = 226). Most respondents reside in southern Italy (53.4%; *N* = 262), followed by central (25.9%; *N* = 127) and northern Italy (20.8%; *N* = 102). The employment profile was mainly constituted by students (45.2%, *N* = 222) and full-time employed (36.3%, *N* = 178). 3.7% (*N* = 18) of respondents declared to have lost their job during the current pandemic. Overall, 50.1% of total respondents declared to be satisfied (26.1%, *N* = 128) or quite satisfied (24%, *N* = 118) regarding their own current financial situation. Only 11.4% (*N* = 56) of the sample declared a pre-existing physical illness before the COVID-19 pandemic, while 5.3% (*N* = 26) were previously affected by a mental disorder. Regarding the COVID-19-related variables, only 4 participants (0.8%) declared a previous COVID-19 infection, 1.8% (*N* = 9) declared to have experienced a home-based isolation due to the COVID-19, and only one subject had been hospitalized due to the COVID-19 infection. About 4.9% (*N* = 24) reported to have been isolated due to the contact with a subject infected by COVID-19 (Table 1).

**Table 1:** Socio-demographic characteristics of the sample

Characteristics	Total sample (*N* = 491)	BSNAS − (*N* = 119)	BSNAS + (*N* = 372)	*p*-values
Age, years, mean ± SD	21.8 ± 1.7	21.6 ± 1.8	21.9 ± 1.7	*t = − 1.374 *p* = 0.183
Gender, % (*N*)				
Female	64.4% (*N* = 316)	27.2% (*N* = 86)	72.8% (*N* = 230)	**χ2(1) = 4.285 *p* = 0.047***
Male	35.6% (*N* = 175)	18.9% (*N* = 33)	81.1% (N = 142)	
Marital status, % (*N*)				
Single	58% (*N* = 285)	16.5% (*N* = 47)	83.5% (*N* = 238)	**χ2(3) = 24.623 ***p* < 0.001**
Married or cohabiting	38.3% (*N* = 188)	33.5% (*N* = 63)	66.5% (*N* = 125)	
Separated or divorced	2.9% (*N* = 14)	50% (*N* = 7)	50% (*N* = 7)	
Widowed	0.8% (*N* = 4)	50% (*N* = 2)	50% (*N* = 2)	
Education level, % (*N*)				
University degree, yes, % (*N*)	53% (*N* = 260)	27.7% (*N* = 72)	72.3% (*N* = 188)	**χ2(3) = 7.121 *p* = 0.068
High school degree, yes, % (*N*)	46% (*N* = 226)	19.9% (*N* = 45)	80.1% (*N* = 181)	
Middle school, yes, % (*N*)	0.8% (*N* = 4)	25% (*N* = 1)	75% (*N* = 3)	
Elementary school, yes, % (*N*)	0.2% (*N* = 1)	100% (*N* = 0)	0 (*N* = 0)	
Employment level, % (*N*)				
Full-time employed, yes, % (*N*)	36.3% (*N *= 178)	32% (*N *= 57)	68% (*N* = 121)	**χ2(4) = 29.422 ***p* < 0.001**
Unemployed, yes, % (*N*)	2.6% (*N* = 13)	23.1% (*N* = 3)	76.9% (*N* = 10)	
Student, yes, % (*N*)	45.2% (*N* = 222)	13.5% (N = 30)	86.5% (*N* = 192)	
Full-time homemaker, yes, % (*N*)	8.8% (*N* = 43)	30.2% (*N* = 13)	69.8% (*N* = 30)	
Lost job due to the pandemic, yes, % (*N*)	3.7% (*N* = 18)	11.1% (*N* = 2)	88.9% (*N* = 16)	**χ2(1) = 1.753 *p* = 0.147***
Any comorbid physical condition(s), yes, % (*N*)	11.4% (*N *= 56)	33.9% (*N* = 19)	66.1% (*N* = 37)	**χ2(1) = 3.234 *p* = 0.072
Any mental health problem(s), yes, % (*N*)	5.3% (*N* = 26)	15.4% (*N* = 4)	84.6% (*N *= 22)	**χ2(1) = 1.171 *p* = 0.279***
Have you been infected by COVID-19, yes, % (*N*)	0.8% (*N* = 4)	25% (*N* = 1)	75% (*N* = 3)	n.v
Have you been isolated due to COVID-19 infection, yes, % (*N*)	1.8% (*N* = 9)	11.1% (*N* = 1)	88.9% (*N* = 8)	n.v
Have you been hospitalized due to COVID-19 infection, yes, % (*N*)	0.2% (*N* = 1)	100% (*N* = 1)	0% (*N* = 0)	n.v
Have you been isolated due to a contact with someone affected by COVID-19, yes, % (*N*)	4.9% (*N* = 24)	25% (*N* = 6)	75% (*N* = 18)	**χ2(1) = 0.008 *p* = 0.546***
Phase II	47.5% (*N* = 233)	26.2% (*N* = 61)	73.8% (*N* = 172)	**χ2(1) = 0.913 *p* = 0.339
Phase III	52.5% (*N* = 258)	22.5% (*N* = 58)	77.5% (*N* = 200)	

*n.v.* not valid

^*^t-Student test

^**^χ2 test

^***^ Fisher’s exact test

Significant differences are in bold

The mean total score of DASS-21 was 60.1 (SD = 12.8), DASS-21 depression subscale was 20.1 (SD = 5.4), DASS-21 anxiety subscale was 23.1 (SD = 4.7), and DASS-21 stress subscale was 16.9 (SD = 5.3) (Table 2). The mean total score of BSNAS was 20.4 (SD = 6.8) with about 75.8% of the sample who were classified as having a PSMU (BSNAS ≥ 16). Interestingly, significantly higher DASS-21 total scores, depression, anxiety and stress levels were found in those participants without clinically significant BSNAS scores (BSNAS− group) compared to BSNAS+ group (all with *p* < 0.001) (Table 2).

**Table 2:** Clinical characteristics of the sample

	Total sample (*N* = 491)	BSNAS − (*N* = 119)	BSNAS + (*N* = 372)	*p*-values
DASS-21 total score, mean ± SD	60.1 ± 12.8	67.0 ± 12.2	57.9 ± 12.2	*t* = 7.100 ***p* < 0.001**
DASS-21, depression subscale, mean ± SD	20.1 ± 5.4	22.3 ± 5.2	19.3 ± 5.3	*t = *5.891 ***p* < 0.001**
DASS-21, anxiety subscale, mean ± SD	23.1 ± 4.7	25.2 ± 3.4	22.4 ± 4.9	*t *= 5.816 ***p *< 0.001**
DASS-21, stress subscale, mean ± SD	16.9 ± 5.3	19.5 ± 5.5	16.1 ± 4.9	*t* = 6.324 ***p* < 0.001**
BIS-15 total score, mean ± SD	39.1 ± 7.6	36.9 ± 7.6	39.7 ± 7.5	*t *= − 3.603 ***p* < 0.001**
BIS-15, attentional Impulsiveness subscale, mean ± SD	11.1 ± 3.5	10.2 ± 3.3	11.3 ± 3.5	*t* = − 3.234 ***p* < 0.001**
BIS-15, motor Impulsiveness subscale, mean ± SD	12.8 ± 4.6	11.7 ± 4.9	13.1 ± 4.4	*t* = − 2.898 ***p* = 0.007**
BIS-15, nonplanning impulsiveness subscale, mean ± SD	10.8 ± 2.4	10.7 ± 2.5	10.8 ± 2.4	*t* = − 0.372 *p* = 0.715
AQ total score, mean ± SD	98.9 ± 15.3	106.9 ± 15.2	96.4 ± 14.4	*t* = 6.882 ***p* < 0.001**
AQ, physical aggression subscale, mean ± SD	34.7 ± 5.0	35.8 ± 4.1	34.3 ± 5.2	*t* = 2.733 ***p* = 0.002**
AQ, verbal aggression subscale, mean ± SD	13.3 ± 3.9	14.5 ± 4.3	12.9 ± 3.7	*t* = 3.812 ***p* < 0.001**
AQ, anger subscale, mean ± SD	25.2 ± 5.0	27.4 ± 5.0	24.6 ± 4.8	*t* = 5.521 ***p* < 0.001**

*AQ* Aggression Questionnaire, *BIS-15* Barratt Impulsiveness Scale-15 items, *BSNAS* Bergen Social Networking Addiction Scale, *BSNAS* + presence of social networking addiction, *BSNAS −* absence of social networking addiction, *DASS-21* depression anxiety stress scale-21 items, *SD* standard deviation

^*^t-Student test

Significant differences are in bold

### Predictors of the problematic social networking use

According to the multivariate regression model, social networking use levels were reduced by the presence of a COVID-19 related hospitalization (Beta coefficient, B = − 12.135; 95% confidence interval, CI = (− 24.038)–(− 0.233)], high levels of AQ total scores [B = − 0.123, 95%CI = (− 0.163)–(− 0.083)] and high levels of general psychiatric symptomatology (as measured by DASS-21 total score) [B = − 0.128, 95%CI = (− 0.175)–(− 0.080)]. These variables statistically significantly predicted social networking use levels (*F* (3, 487) = 42.338, *p* < 0.001, *R*^2^ = 0.207).

**Fig. 1:**
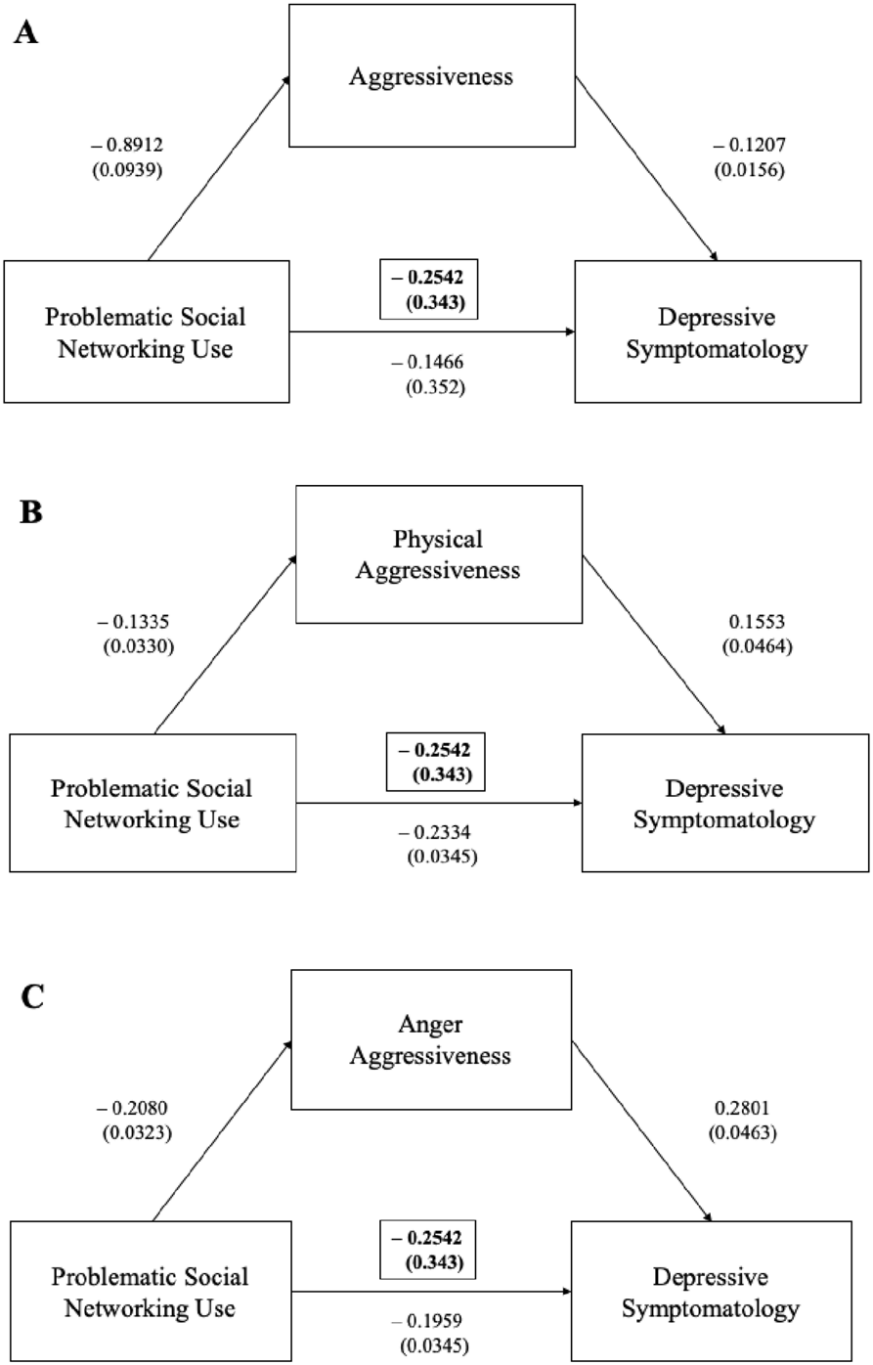
Aggressiveness in the mediation analyses between the problematic social networking use and depressive symptomatology

**Fig. 2:**
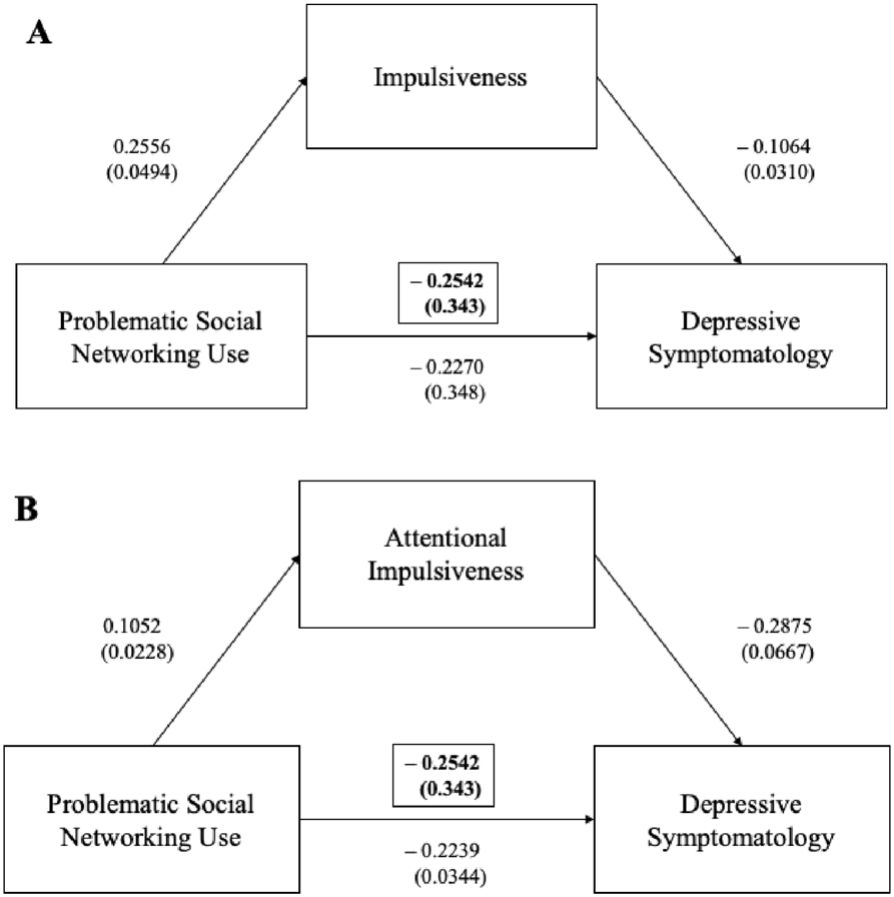
Impulsiveness in the mediation analyses between the problematic social networking use and depressive symptomatology

Mediation analyses showed that social networking use (as measured by BSNAS) negatively predicted depressive symptomatology and that their interaction is mediated by AQ total score (*ß* = − 0.1075, 95%CI [(− 0.1449)–(− 0.0740)]) (Fig. 1A); AQ physical aggression subscale (*ß* = − 0.207, 95%CI [(− 0.378)–(− 0.0069)]) (Fig. 1B); AQ anger subscale (*ß* = − 0.0582, 95%CI [(− 0.0863–0.0351]) (Fig. 1C); BIS-11 total score (*ß* = − 0.0272, 95%CI [(− 0.0482)–(− 0.0100)]) (Fig. 2A), and BIS-11 attentional subscale (*ß* = − 0.0302, 95%CI [(− 0.0520)–(− 0.0130)]) (Fig. 2B).

Legend Fig. 1 Mediation analyses showed that the problematic social networking use negatively predicted depressive symptomatology and that their interaction was mediated by aggressiveness total score (Fig. 1A), physical aggression score (Fig. 1B) and anger/aggressiveness subscale (Fig. 1C).

Legend Fig. 2 Mediation analysis showed that the problematic social networking use negatively predicted depressive symptomatology and that their interaction was mediated by impulsiveness total score (Fig. 2A), attentional impulsiveness (Fig. 2B).

### Predictors of the COVID-19-related psychopathology

According to the multivariate regression model, high COVID-19-related depressive levels were predicted by high levels of AQ total scores (*B* = 0.218, 95%CI = 0.165–0.272, *p* < 0.001). On the contrary, depressive levels were reduced by the presence of a mental disorder [*B* = − 3.692, 95%CI = − 5535)–(− 0.189), *p* < 0.001], high levels of BSNAS [*B* = − 0.119, 95%CI = (-0.186)–(− 0.052), *p* < 0.001], high levels of verbal aggressiveness [*B* = − 0.338, 95%CI = (− 0.491)–(− 0.185), *p* < 0.001], high levels of physical aggressiveness [*B* = − 0.207, 95%CI = (− 0.324)–(− 0.090), *p* < 0.001] and high levels of attentional impulsiveness [*B* = − 0.153, 95%CI = (− 0.278)–(− 0.029), *p* = 0.016], which significantly predicted depressive levels *F*(6, 484) = 30.322, *p* < 0.001, *R*^2^ = 0.273 (Table 3).

**Table 3:** Multinomial linear regression model (outcome = depression DASS-21 subscale)

	***B***	**SE**	*ß*	***t***	***p*-value (two-tailed)**
(constant)	18.122	2.393		7.573	< 0.001
AQ total score	0.218	0.027	0.618	8.020	** < 0.001**
Mental disorder (yes)	− 3.692	0.938	− 0.153	− 3.936	** < 0.001**
BSNAS total score	− 0.119	0.034	− 0.149	− 3.477	** < 0.001**
AQ verbal aggression	− 0.338	0.078	− 0.244	− 4.342	** < 0.001**
AQ physical aggression	− 0.207	0.060	− 0.192	− 3.473	** < 0.001**
BIS-15 attentional	− 0.153	0.063	− 0.099	− 2.419	**0.016**

*AQ* Aggression Questionnaire, *BIS-15* Barratt Impulsiveness Scale-15 items, *BSNAS* Bergen Social Networking Addiction Scale

Significant values are in bold

High anxiety levels were predicted by high levels of AQ total scores (*B* = 0.146, 95%CI = 0.097–0.195, *p* < 0.001). Anxiety levels were reduced by the presence of a mental disorder [*B* = − 2.153, 95%CI = (− 3.837)–(− 0.470), *p* = 0.012], high levels of BSNAS [*B* = − 0.103, 95%CI = (− 0.164)–(− 0.042), *p* = 0.001], high levels of verbal aggressiveness [*B* = − 0.213, 95%CI = (− 0.353)–(− 0.074), *p* = 0.003], high levels of physical aggressiveness [*B* = − 0.116, 95%CI = (− 0.223)–(− 0.009),− 0.253)–(− 0.025), *p* = 0.017], which significantly predicted depressive levels, *F* (6, 484) = 19.944, *p* < 0.001, *R*^2^ = 0.198.

High stress levels were predicted by high levels of AQ total scores (*B* = 0.192, 95%CI = 0.138–0.246, *p* < 0.001). Stress levels were reduced by high levels of BSNAS [*B* = − 0.126, 95% CI = (− 0.194)–(− 0.059), *p* < 0.001], high levels of verbal aggressiveness [*B* = − 0.250, 95%CI = (− 0.403)–(− 0.097), *p* = 0.001], high levels of physical aggressiveness (*B* = − 0.216, 95%CI = (− 0.333)–(− 0.098), *p* < 0.001] and high levels of attentional impulsiveness [*B* = − 0.151, 95%CI = (− 0.276)–(− 0.026), *p* = 0.018], which significantly predicted depressive levels, *F* (5, 485) = 28.062, *p* < 0.001, *R*^2^ = 0.224.

Furthermore, general psychopathology was predicted by high levels of AQ total scores (*B* = 0.556, 95%CI = 0.434–0.678, *p* < 0.001) and reduced by the presence of a mental disorder [*B* = − 5.962, 95%CI = (− 10.169)–(− 1.755), *p* = 0.006], high levels of BSMAS [*B* = − 0.348, 95%CI = (− 0.501)–(− 0.195), *p* < 0.001], high levels of verbal aggressiveness [*B* = − 0.801, 95%CI = (− 1.150)–(− 0.452), *p* < 0.001], high levels of physical aggressiveness [*B* = − 0.539, 95%CI = (− 0.806)–(− 0.271), *p* < 0.001] and high levels of attentional impulsiveness [*B* = − 0.443, 95%CI = (− 0.727)–(− 0.159), *p* = 0.002]. These variables statistically significantly predicted depressive levels, *F* (6, 484) = 39.201, *p* < 0.001, *R*^2^ = 0.327.

**Fig. 3:**
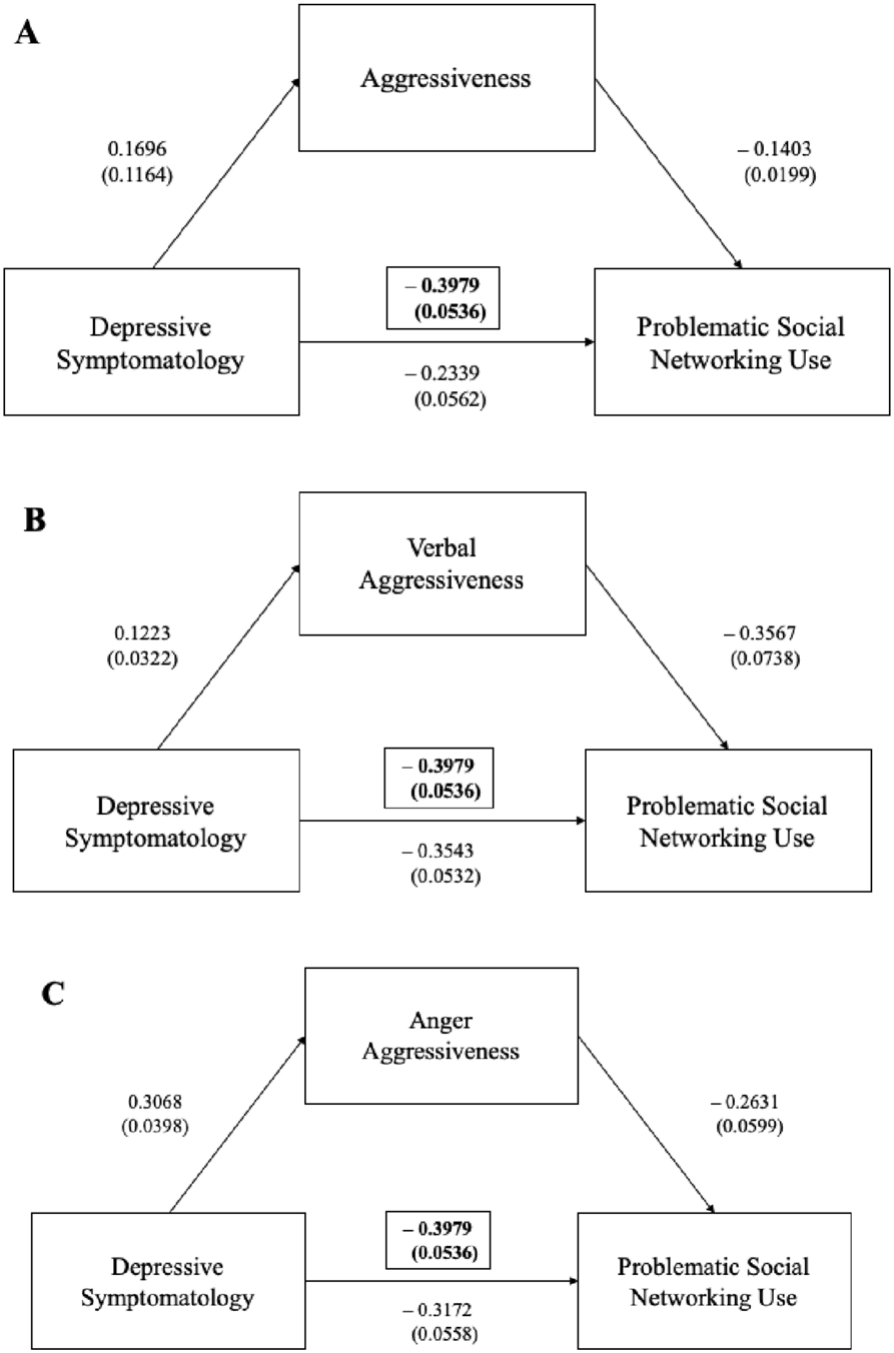
Aggressiveness in the mediation analyses between the depressive symptomatology and problematic social networking use

**Fig. 4:**
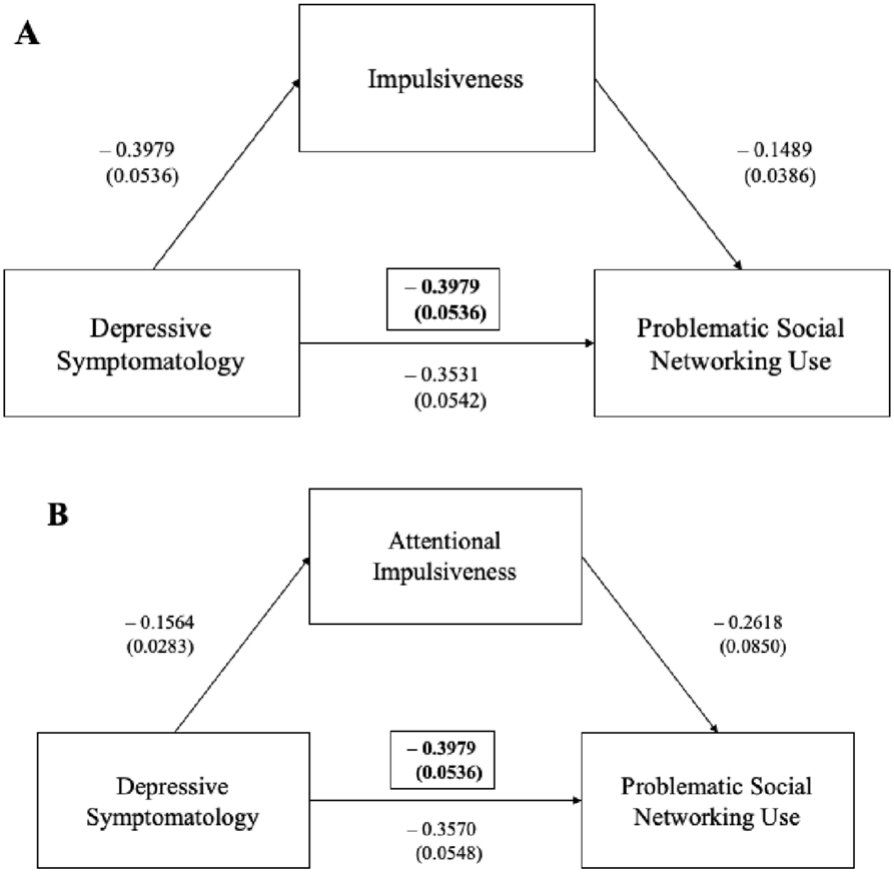
Impulsiveness in the mediation analyses between the depressive symptomatology and problematic social networking use

Mediation analyses showed that COVID-19 related depressive symptomatology significantly predicted social networking use (as measured by BSNAS) and that their interaction is negatively mediated by AQ total score (*ß* = − 0.1640, 95%CI [(− 0.2237)–(− 0.1130)]) (Fig. 3A), AQ verbal aggression subscale (*ß* = 0.0436, 95%CI [(0.0760)–(0.0178)]) (Fig. 3B), AQ anger subscale (*ß* = -0.0807, 95%CI [(− 0.1237–0.0431]) (Fig. 3C), BIS-11 total score (*ß* = − 0.0448, 95%CI [(− 0.0771)–(− 0.0193)]) (Fig. 4A), and BIS-11 attentional subscale (*ß* = − 0.0409, 95%CI [(− 0.0721)–(− 0.0153)]) (Fig. 4B).

Legend Fig. 3 Mediation analyses showed that COVID-19-related depressive symptomatology significantly predicted social networking use and that their interaction was negatively mediated by aggressiveness (Fig. 3A), verbal aggression (Fig. 3B), and anger (Fig. 3C).

Legend Fig. 4 Mediation analyses showed that COVID-19 related depressive symptomatology significantly predicted social networking use and that their interaction was negatively mediated by impulsiveness (Fig. 4A) and attentional impulsiveness (Fig. 4B).

## Discussion

Social media use has been supposed to cover a pathoplastic role in the development of psychological impairment and/or psychiatric disorders [30–33]. However, it is still controversial the impact of SM use on wellbeing, life satisfaction and mental illness. Actually, such a complex interaction might be influenced by both internal and external factors, including social isolation, decreased community engagement, and loneliness [26, 60–63]. In turn, the impact of SM use on mental wellbeing/illness is influenced by several factors such as use modality (occasional vs. excessive), users’ age and gender, intrinsic and extrinsic motivations in SM use (e.g., to manage impressions, to share emotions, to reduce loneliness feelings, to increase social connectedness) [64, 65].

Our findings show a higher SM use among young people, with 75.8% of them who were classified as having a PSMU (as measured by BSNAS). Interestingly, significantly higher DASS-21 total scores, depression, anxiety and stress levels were found in those participants without clinically significant BSNAS scores; while subjects with PSMU did not manifest a significant psychopathological burden. Our findings point to a ‘protective/resilient’ role of social networking use in mitigating the depressive symptomatology in people aged 18–24, during the early phases of the COVID-19 outbreak. The effect did not change, after controlling for the type of COVID-19 phase (II vs. III), gender, and all COVID-19 disease-related variables. Our findings are in line with previous studies that found a positive effect of SM use in overcoming the negative consequences of COVID-19-related social isolation, by reducing loneliness and increasing social connectedness [66–68]. However, other studies reported a detrimental association, by outlining that COVID-19-related restrictive measures and lockdown favored an excessive SM use and increased the risk of developing a PSMU [39, 69–71]. Indeed, a recent metanalysis collecting data coming from 14 cross-sectional studies carried out during the COVID-19 pandemic among young people in different countries, reported that an excessive time spent on social networking platforms was much more likely associated with an increased risk of developing depressive (OR = 1.43) and anxiety symptoms (OR = 1.55) [72].

Studies carried out before COVID-19 often reported inconclusive and contrasting results about the link between SM use and depression [18, 23, 28, 73–77], probably due the correlational nature of most studies that precludes causal inference. Some authors proposed a bidirectional association, by supporting the hypothesis that PSMU may lead to depression and, similarly, depression may increase a pre-existing PSMU [78]. However, another systematic review found that the time spent by young people on SM was significantly associated with the occurrence of depressive, anxiety and psychological distress symptoms, also in those without a previous mental distress and/or psychiatric disorder [28]. A more recent “dose–response” meta-analysis demonstrated a linear dose–response and gender-specific association between time spent on SM and the risk of depression in young people (OR = 1.6), with a stronger association mainly found in females (OR = 1.7) versus males (OR = 1.20) [79]. Thus, one could argue that the time spent on SM would be the key to discriminate between a protective versus detrimental effect, even though further studies should confirm this hypothesis.

Our findings could then be interpreted considering that forced online activities experienced by the general population, during the early COVID-19 phases, could have potentially protected young people by the emergence of loneliness, anxiety, fear for the future, and depressive symptoms. As our study collected data during the second and third COVID-19 Italian wave [43], we were able only to partially describe the effect of the first COVID-19-related Italian lockdown and did not weight the cumulative effect of the COVID-19 situation over the time. However, other studies carried out under the COVID-19-related restrictions reported that SM use helped to maintain a close emotional bond with distant family members, friends and peers, by facilitating the acceptance of the required condition to stay-at-home as well as favoring to overcome experienced loneliness and social isolation [80]. Our findings suggest that this protective effect refers mainly to the younger population, who could find beneficial use of social networking to remain in contact with their peers and friends. Therefore, one could argue that this association could be particularly relevant for the digital natives’ generation and probably only in the early stages of the COVID-19 outbreak. Merchant and Lurie [80] found that temporary communication means, substituted by the lack of the physical contact (e.g., with friends, acquaintances, etc.) by social networking platforms, in times of the COVID-19 pandemic, are considered useful to satisfy individuals’ needs for disaster-related information, entertainment as well as for maintaining interpersonal communication [80]. A recent prospective 9-month follow-up cohort study, investigating the association between SM use and risk of depression in Chinese adolescents during COVID-19 pandemic, reported that the protective effect of online social networking use for depression was not maintained over the time, suggesting a chronological trend [79].

According to our findings, SM use could be a potential coping strategy adopted by digital natives in response to stressful life events, due to the COVID-19-related restrictions. Indeed, Lazarus and Folkman [81] proposed two main types of coping strategies to manage stressful events: (a) problem-focused coping (i.e., engage in behaviors that could help solve problems); and (b) the emotion-focused coping (i.e., regulate emotional responses to the problem without affecting the actual presence of stress) [82]. Within this theoretical model, the challenges determined by the COVID-19 situation may have forced young people to more likely turn to social networking for both problem-focused coping (e.g., browsing health-related information) and emotion-focused coping (e.g., venting emotions for mood management, joining online communities for social support, meeting online friends and peers) [83]. Accordingly, our findings found that young people experiencing a PSMU significantly described lower aggressiveness (physical and verbal) and anger levels compared to those without a PSMU, who reported significantly higher levels of general psychopathology, anxiety, stress and depression. Indeed, our PSMU sample reported significantly higher impulsiveness levels (particularly, motor and attentional impulsiveness) compared to young people without a PSMU. Our mediation analyses also demonstrated that the association between higher depression levels and lower BSNAS levels was mediated by higher aggressiveness and lower impulsiveness levels. Therefore, the protective effect of social networking use could be beneficial to younger people, at different degrees according to specific personality profiles (i.e., levels of impulsiveness and aggressiveness).

Future studies should confirm these findings and evaluate specifically the role of the impulsiveness and aggressiveness personality traits in mediating the relationship between PSMU and depression, along stressful events. Furthermore, our study contains limitations that should be considered before generalizing our findings. First, the cross-sectional design allows only hypothetical conclusions of causality between the protective role of SM use in the emergence of depressive symptomatology in the general population during COVID-19 outbreak. We could not argue whether the SM use was protective only during the early stages of COVID-19 phases and if such a protective effect will be maintained over time. Therefore, longitudinal studies should collect data, considering the number of occurring COVID-19 lockdowns, the intensity and frequency in social networking use pre-COVID-19, during each COVID-19-related phase and post-COVID-19. Second, the convenience sampling method and online recruitment strategy, while allowed to overcome the recruitment obstacles due to physical distancing measures, could be highly vulnerable to selection bias and may imply a significant sampling problem. Third, our sample included apparently healthy participants (without a previous PSMU) and, to validate the association between SM use and depression, our findings should be replicated in a clinical sample. Fourth, our sample is mainly constituted by young university students, with a possible unbalance with respect to young workers; thus, further studies should recruit by stratifying workers and students, in order to more reliably compare findings coming from both groups and evaluate whether this protective effect is only found in the university students. Finally, we did not collect data on the time spent on social networking platforms nor regarding the type of social network, therefore, further studies should find out the role of time spent on SM and of the type of used/preferred social networking platform.

Overall, our study documented a potential positive role of social networking platforms, at least during the early stages of the COVID-19 pandemic, particularly in overcoming depressive symptomatology and general psychopathological burden due to the COVID-19 situation, in young adults. Furthermore, our findings highlight the need of implementing youth-friendly targeted SM-based interventions, specifically addressed to digital natives to overcome the emergence of potential depressive symptomatology due to stressful events.

## Data Availability

The datasets used and/or analyzed during the current study are available from the corresponding author on reasonable request.

## References

[1] Kaplan AM, Haenlein M (2010). Users of the world, unite! The challenges and opportunities of social media. Bus Horiz.

[2] Torous J, Bucci S, Bell IH, Kessing LV, Faurholt-Jepsen M, Whelan P, Carvalho AF, Keshavan M, Linardon J, Firth J (2021). The growing field of digital psychiatry: current evidence and the future of apps, social media, chatbots, and virtual reality. World Psychiatry.

[3] Torous J, Choudhury T, Barnett I, Keshavan M, Kane J (2020). Smartphone relapse prediction in serious mental illness: a pathway towards personalized preventive care. World Psychiatry.

[4] Griths MD, Kuss DJ, Demetrovics Z, Rosenberg KP, Feder LC (2014). Social networking addiction: An overview of preliminary findings. Behavioral addictions.

[5] Rideout V, Fox S. Digital Health Practices, Social Media Use, and Mental Well-Being Among Teens and Young Adults in the US. Hopelab and Well Being Trust. 2018. Available at: https://digitalcommons.psjhealth.org/cgi/viewcontent.cgi?article=2092&context=publications. Accessed 1 Sept 2022.

[6] Yellow. Yellow social media report 2018: part one consumers. 2018. Available at: https://www.yellow.com.au/wp-content/uploads/2018/06/Yellow-Social-Media-Report-2018-Consumer.pdf. Accessed 1 Sept 2022.

[7] Holt-Lunstad J (2021). A pandemic of social isolation?. World Psychiatry.

[8] Liu D, Baumeister RF (2016). Social networking online and personality of self-worth: a meta-analysis. J Res Personal.

[9] Burrow AL, Rainone N (2017). How many likes did I get? Purpose moderates links between positive social media feedback and self-esteem. J Exp Soc Psychol.

[10] Ryan T, Allen K, Gray D, McInernery D (2017). How social are social media? A review of online social behaviour and connectedness. J Relatsh Res.

[11] Verduyn P, Gugushvili N, Kross E (2021). The impact of social network sites on mental health: distinguishing active from passive use. World Psychiatry.

[12] Smith NR, Clark C, Smuk M, Cummins S, Stansfeld SA (2015). The influence of social support on ethnic differences in well-being and depression in adolescents: findings from the prospective Olympic Regeneration in East London (ORiEL) study. Soc Psychiatry Psychiatr Epidemiol.

[13] He F, Zhou Q, Li J, Cao R, Guan H (2014). Effect of social support on depression of internet addicts and the mediating role of loneliness. Int J Ment Health Syst.

[14] Liu HY, Beresin EV, Chisolm MS (2019). Social media skills for professional development in psychiatry and medicine. Psychiatr Clin North Am.

[15] Andreassen CS (2015). Online social network site addiction: a comprehensive review. Curr Addict Rep.

[16] Andreassen CS, Pallesen S (2014). Social network site addiction—an overview. Curr Pharm Des.

[17] Andreassen CS, Pallesen S, Griffiths MD (2017). The relationship between addictive use of social media, narcissism, and self-esteem: findings from a large national survey. Addict Behav.

[18] Hussain Z, Griffiths MD (2018). Problematic social networking site use and comorbid psychiatric disorders: a systematic review of recent large-scale studies. Front Psychiatry.

[19] Warburton WA, Strasburger VE (2021). Should internet addiction and gaming addiction be categorized as disorders. Masters of media: controversies and solutions.

[20] Meng SQ, Cheng JL, Li YY, Yang XQ, Zheng JW, Chang XW (2022). Global prevalence of digital addiction in general population: a systematic review and meta-analysis. Clin Psychol Rev.

[21] Liu QQ, Zhou ZK, Yang XJ, Niu GF, Tian Y, Fan CY (2017). Upward social comparison on social network sites and depressive symptoms: a moderated mediation model of self-esteem and optimism. Personal Individ Differ.

[22] Gerber JP, Wheeler L, Suls J (2018). A social comparison theory meta-analysis 60+ years on. Psychol Bull.

[23] Yoon S, Kleinman M, Mertz J, Brannick M (2019). Is social network site usage related to depression? A meta-analysis of facebook-depression relations. J Affect Disord.

[24] Appel M, Marker C, Gnambs T (2020). Are social media ruining our lives?. Rev Gen Psychol.

[25] Huang C (2021). Correlations of online social network size with well-being and distress: a meta-analysis. Cyberpsychol: J Psychosoc Res Cyberspace.

[26] Samra A, Warburton WA, Collins AM (2022). Social comparisons: a potential mechanism linking problematic social media use with depression. J Behav Addict.

[27] Shensa A, Sidani JE, Dew MA, Escobar-Viera CG, Primack BA (2018). Social media use and depression and anxiety symptoms: a cluster analysis. Am J Health Behavior.

[28] Keles B, McGrae N, Grealish A (2020). A systematic review: the influence of social media on depression, anxiety and psychological distress in adolescents. Int J Adolesc Youth.

[29] Huang C (2022). A meta-analysis of the problematic social media use and mental health. Int J Soc Psychiatry.

[30] Orben A (2020). Teenagers, screens and social media: a narrative review of reviews and key studies. Soc Psychiatry Psychiatr Epidemiol.

[31] Alonzo R, Hussain J, Stranges S, Anderson KK (2021). Interplay between social media use, sleep quality, and mental health in youth: a systematic review. Sleep Med Rev.

[32] Girela-Serrano BM, Spiers ADV, Ruotong L, Gangadia S, Toledano MB, Di Simplicio M (2022). Impact of mobile phones and wireless devices use on children and adolescents’ mental health: a systematic review. Eur Child Adolesc Psychiatry.

[33] Abi-Jaoude E, Naylor KT, Pignatiello A (2020). Smartphones, social media use and youth mental health. CMAJ.

[34] Sohrabi C, Alsafi Z, O'Neill N, Khan M, Kerwan A, Al-Jabir A (2020). World health organization declares global emergency: a review of the 2019 novel coronavirus (COVID-19). Int J Surg.

[35] Greenberg N, Rafferty L (2021). Post-traumatic stress disorder in the aftermath of COVID-19 pandemic. World Psychiatry.

[36] Unützer J, Kimmel RJ, Snowden M (2020). Psychiatry in the age of COVID-19. World Psychiatry.

[37] Adhanom GT (2020). Addressing mental health needs: an integral part of COVID-19 response. World Psychiatry.

[38] Bayer JB, Anderson IA, Tokunaga RS (2022). Building and breaking social media habits. Curr Opin Psychol.

[39] Brailovskaia J, Margraf J (2021). The relationship between burden caused by coronavirus (Covid-19), addictive social media use, sense of control and anxiety. Comput Hum Behav.

[40] Gao J, Zheng P, Jia Y, Chen H, Mao Y, Chen S (2020). Mental health problems and social media exposure during COVID-19 outbreak. PLoS ONE.

[41] Fiorillo A, Sampogna G, Giallonardo V, Del Vecchio V, Luciano M, Albert U (2020). Effects of the lockdown on the mental health of the general population during the COVID-19 pandemic in Italy: results from the COMET collaborative network. Eur Psychiatry.

[42] Giallonardo V, Sampogna G, Del Vecchio V, Luciano M, Albert U, Carmassi C (2020). The impact of quarantine and physical distancing following COVID-19 on mental health: study protocol of a multicentric Italian population trial. Front Psychiatry.

[43] Volpe U, Orsolini L, Salvi V, Albert U, Carmassi C, Carra G (2022). COVID-19-related social isolation predispose to problematic internet and online video gaming use in Italy. Int J Environ Res Public Health.

[44] Luo T, Chen W, Liao Y (2021). Social media use in China before and during COVID-19: preliminary results from an online retrospective survey. J Psychiatr Res.

[45] Marengo D, Angelo Fabris M, Longobardi C, Settanni M (2022). Smartphone and social media use contributed to individual tendencies towards social media addiction in Italian adolescents during the COVID-19 pandemic. Addict Behav.

[46] Fousiani K, Dimitropoulou P, Michaelides MP, Van Petegem S (2016). Perceived parenting and adolescent cyber-bullying: examining the intervening role of autonomy and relatedness need satisfaction, empathic concern and recognition of humanness. J Child Fam Stud.

[47] Hou Y, Xiong D, Jiang T, Song L, Wang Q (2019). Social media addiction: Its impact, mediation, and intervention. Cyberpsychol: J Psychosoc Res Cyberspace.

[48] Li JB, Mo PKH, Lau JTF, Su XF, Zhang X, Wu AMS (2018). Online social networking addiction and depression: The results from a large-scale prospective cohort study in Chinese adolescents. J Behav Addict.

[49] Cheng C, Lau YC (2022). Social media addiction during COVID-19-mandated physical distancing: relatedness needs as motives. Int J Environ Res Public Health.

[50] World Health Organization (WHO) (2020). Adolescent mental health.

[51] Bottesi G, Ghisi M, Altoe G, Conforti E, Melli G, Sica C (2015). The Italian version of the depression anxiety stress scales-21: factor structure and psychometric properties on community and clinical samples. Compr Psychiatry.

[52] Lovibond PF, Lovibond SH (1995). The structure of negative emotional states: comparison of the depression anxiety stress scales (dass) with the beck depression and anxiety inventories. Behav Res Ther.

[53] Monacis L, de Palo V, Griffiths MD, Sinatra M (2017). Social networking addiction, attachment style, and validation of the Italian version of the bergen social media addiction scale. J Behav Addict.

[54] Andreassen CS, Pallesen S, Griffiths MD, Torsheim T, Sinha R (2018). The development and validation of the bergen-yale sex addiction scale with a large national sample. Front Psychol.

[55] Orozco-Cabal L, Rodriguez M, Herin DV, Gempeler J, Uribe M (2010). Validity and reliability of the abbreviated barratt impulsiveness scale in Spanish (BIS-15S). Rev Colomb Psiquiatr.

[56] Patton JH, Stanford MS, Barratt ES (1995). Factor structure of the Barratt impulsiveness scale. J Clin Psychol.

[57] Spinella M (2007). Normative data and a short form of the Barratt impulsiveness scale. Int J Neurosci.

[58] Buss AH, Perry MP (1992). The aggression questionnaire. J Pers Soc Psychol.

[59] Hayes AF, Preacher KJ (2014). Statistical mediation analysis with a multicategorical independent variable. Br J Math Stat Psychol.

[60] Wegmann E, Stodt B, Brand M (2015). Addictive use of social networking sites can be explained by the interaction of Internet use expectancies, Internet literacy, and psychopathological symptoms. J Behav Addict.

[61] Baker DA, Algorta GP (2016). The relationship between online social networking and depression: a systematic review of quantitative studies. Cyberpsychol Behav Soc Netw.

[62] Frost RL, Rickwood DJ (2017). A systematic review of the mental health outcomes associated with Facebook use. Comput Hum Behav.

[63] Shannon H, Bush K, Villeneuve PJ, Hellemans KG, Guimond S (2022). Problematic social media use in adolescents and young adults: systematic review and meta-analysis. JMIR Ment Health.

[64] Montag C, Hegelich S, Sindermann C, Rozgonjuk D, Marengo D, Elhai JD (2021). On corporate responsibility when studying social media use and well-being. Trends Cogn Sci.

[65] Rogowska AM, Libera P (2022). life satisfaction and instagram addiction among university students during the COVID-19 pandemic: the bidirectional mediating role of loneliness. Int J Environ Res Public Health.

[66] Marzouki YAFS, Veltri GA (2021). Understanding the buffering effect of social media use on anxiety during the COVID-19 pandemic lockdown. Humanit Soc Sci Commun.

[67] Ruggieri S, Ingoglia S, Bonfanti RC, Lo CG (2021). The role of online social comparison as a protective factor for psychological wellbeing: a longitudinal study during the COVID-19 quarantine. Pers Individ Dif.

[68] Di Blasi M, Salerno L, Albano G, Caci B, Esposito G, Salcuni S (2022). A three-wave panel study on longitudinal relations between problematic social media use and psychological distress during the COVID-19 pandemic. Addict Behav.

[69] Boursier V, Gioia F, Musetti A, Schimmenti A (2020). Facing loneliness and anxiety during the COVID-19 isolation: the role of excessive social media use in a sample of italian adults. Front Psychiatry.

[70] Geirdal AO, Ruffolo M, Leung J, Thygesen H, Price D, Bonsaksen T (2021). Mental health, quality of life, wellbeing, loneliness and use of social media in a time of social distancing during the COVID-19 outbreak. a cross-country comparative study. J Ment Health.

[71] Zhao N, Zhou G (2021). COVID-19 stress and addictive social media use (smu): mediating role of active use and social media flow. Front Psychiatry.

[72] Lee Y, Jeon YJ, Kang S, Shin JI, Jung YC, Jung SJ (2022). Social media use and mental health during the COVID-19 pandemic in young adults: a meta-analysis of 14 cross-sectional studies. BMC Public Health.

[73] McCrae N, Gettings S, Purssell E (2017). Social media and depressive symptoms in childhood and adolescence: a systematic review. Adolesc Res Rev.

[74] Ivie EJ, Pettitt A, Moses LJ, Allen NB (2020). A meta-analysis of the association between adolescent social media use and depressive symptoms. J Affect Disord.

[75] Stewart DE, Appelbaum PS (2020). COVID-19 and psychiatrists' responsibilities: a WPA position paper. World Psychiatry.

[76] Stein DJ, Craske MG, Rothbaum BO, Chamberlain SR, Fineberg NA, Choi KW, de Jonge P, Baldwin DS, Maj M (2021). The clinical characterization of the adult patient with an anxiety or related disorder aimed at personalization of management. World Psychiatry.

[77] Maj M, Stein DJ, Parker G, Zimmerman M, Fava GA, De Hert M, Demyttenaere K, McIntyre RS, Widiger T, Wittchen HU (2020). The clinical characterization of the adult patient with depression aimed at personalization of management. World Psychiatry.

[78] Raudsepp L, Kais K (2019). Longitudinal associations between problematic social media use and depressive symptoms in adolescent girls. Prev Med Rep.

[79] Liu M, Kamper-DeMarco KE, Zhang J, Xiao J, Dong D, Xue P (2022). Time spent on social media and risk of depression in adolescents: a dose-response meta-analysis. Int J Environ Res Public Health.

[80] Merchant RM, Lurie N (2020). Social media and emergency preparedness in response to novel coronavirus. JAMA.

[81] Lazarus RS, Folkman S (1984). Stress, appraisal, and coping.

[82] Schoenmakers EC, van Tilburg TG, Fokkema T (2015). Problem-focused and emotion-focused coping options and loneliness: how are they related?. Eur J Ageing.

[83] Van Ingen EUS, Toepoel V (2015). Online coping after negative life events: measurement, prevalence, and relation with Internet activities and well-being. Soc Sci Comput Rev.

